# Age Differences in the Transfer and Maintenance of Practice-Induced Improvements in Task Switching: The Impact of Working-Memory and Inhibition Demands

**DOI:** 10.3389/fpsyg.2017.00410

**Published:** 2017-03-17

**Authors:** Jutta Kray, Balázs Fehér

**Affiliations:** Department of Psychology, Saarland UniversitySaarbruecken, Germany

**Keywords:** task-switching training, transfer, aging, working memory, inhibition

## Abstract

Recent aging studies on training in task switching found that older adults showed larger improvements to an untrained switching task as younger adults do. However, less clear is what type of cognitive control processes can explain these training gains as participants were trained with a particular type of switching task including bivalent stimuli, requiring high inhibition demands, and no task cues helping them keeping track of the task sequence, and by this, requiring high working-memory (WM) demands. The aims of this study were first to specify whether inhibition, WM, or switching demands are critical for the occurrence of transfer and whether this transfer depends on the degree of overlap between training and transfer situation; and second to assess whether practiced-induced gains in task switching can be maintained over a longer period of time. To this end, we created five training conditions that varied in switching (switching vs. single task training), inhibition (switching training with bivalent or univalent stimuli), and WM demands (switching training with or without task cues). We investigated 81 younger adults and 82 older adults with a pretest-training-posttest design and a follow-up measurement after 6 months. Results indicated that all training and age groups showed improvements in task switching and a differential effect of training condition on improvements to an untrained switching task in younger and older adults. For younger adults, we found larger improvements in task switching for the switching groups than the single-task training group independently of inhibition and WM demands, suggesting that practice in switching is most critical. However, these benefits disappeared after 6 months. In contrast, for older adults training groups practicing task switching under high inhibition demands showed larger improvements to untrained switching tasks than the other groups. Moreover, these benefits were maintained over time. We also found that the transfer of benefits in task switching was larger with greater overlap between training and transfer situation. However, results revealed no evidence for transfer to other untrained cognitive task. Overall, the findings suggest that training in resolving interference while switching between two tasks is most critical for the occurrence of transfer in the elderly.

## Introduction

During the last century, life expectancy has increased and this trend is expected to continue in the future (Vaupel, 2010). As a consequence, the relative proportion of individuals above 60 years of age will dramatically increase in the next decades. At the same time it is well documented that aging is associated with substantial decline in many areas of cognitive functioning (for recent reviews, Nyberg et al., 2012; Hartshorne and Germine, 2015). However, the ability to improve cognitive functioning remains considerably intact throughout the adult lifespan (for reviews, Lövdén et al., 2010; Lindenberger, 2014). Therefore, one important challenge for aging researchers is to identify whether and how decline in cognitive functioning can be prevented, maintained, or even reversed through effective training interventions (e.g., Mayr, 2008; Kühn and Lindenberger, 2016). An effective training intervention should not only show that (a) the trained ability can be improved after the intervention, but also determine the extent to which these training gains (b) generalize to other domains of functioning and (c) can be maintained over a longer period of time, and finally (d) what training conditions are the best to promote cognitive plasticity for specific age ranges (e.g., Karbach and Kray, 2009; Kray and Ferdinand, 2014). In this study, we examined all four aspects in order to replicate and extend previous findings on the effectiveness of training in task switching in groups of younger and older adults (cf. Karbach and Kray, 2009). In particular, we aimed at investigating the impact of working-memory (WM) and inhibition demands on practice-related improvements in task switching and their effects on the generalizability to similar and dissimilar cognitive control tasks and on the maintenance over half a year compared to initial task performance.

In our previous training study by Karbach and Kray (2009) we used a task-switching training in order to enhance cognitive control abilities. In this type of training, participants had to switch regularly between two task sets, such as categorizing pictures according to colors (task A) or shapes (task B). Cognitive control is indexed by two types of task-switching costs – here termed mixing and switching costs. Mixing costs are defined as the difference in performance between single-task blocks and mixed-task blocks, whereas switching costs are measured as the difference in performance between switch and non-switch trials within mixed-task blocks (cf. Kray and Lindenberger, 2000). Previous research indicated that age differences are much larger in mixing than in switching costs (for a meta-analysis, see Wasylyshyn et al., 2011) and that age differences in mixing costs are maximized in the absence of task cues and under high ambiguity (for a review, see Kray and Ferdinand, 2014). Therefore, Karbach and Kray (2009) used a particular variant of the task-switching paradigm namely the so-called alternating-runs task-switching (AR-TS) paradigm (for reviews, see Kiesel et al., 2010; Grange and Houghton, 2014). Here participants are instructed to alternate between two tasks within a block, according to a predefined sequence, such as to switch the task on every second trial. Hence, no task cue indicated the next to be performed tasks and participants needed to keep track of the task sequence throughout a block. Furthermore, all stimuli were bivalent (or ambiguous) meaning that all stimuli consisted of features relevant for each of the two tasks and responses of both tasks were partly mapped onto the same response button (cf. Rogers and Monsell, 1995). In order to identify optimal training conditions for different age ranges (i.e., children, younger, and older adults) we compared four different training groups: The active control group only performed the single tasks A or B, while the four treatment groups only performed the alternating-run blocks (task-switching training). The first treatment group only practiced the switching between two tasks; the second treatment group practiced task switching and in addition verbalized the next to be performed task, as verbalization has been found to reduce age differences in mixing costs (cf. Kray et al., 2008). Finally, the third treatment group also practiced task switching with verbalization but received a new set of stimuli in each of the practice sessions, inducing variability during the training that in particular has been found to promote transfer of training (cf. Kramer et al., 1995; Green and Bavelier, 2008).

To examine age differences in the transfer of the task-switching training participants performed untrained but structurally similar switching tasks (referred to as near transfer) and a comprehensive cognitive test battery including two or three indicator tests measuring verbal and visual working memory, inhibition, and fluid intelligence. The results of this training study indicated (a) training-related improvements in task switching in all three age groups; (b) a reduction of mixing and switching costs from pre- to post-test, that is, near transfer gains to a similar switching task that were even more pronounced for children and older adults; (c) and performance improvements in inhibition, working memory, and fluid intelligence in all age groups, suggesting relatively broad far transfer of the switching training (see, Karbach and Kray, 2009). One explanation for this broad transfer effect is that a specific variant of the task-switching paradigm was applied that involved not only practice in switching processes *per se* but also WM processes as subjects had to keep track of the task sequence and inhibition processes as they practiced with bivalent (ambiguous) stimuli.

In the meanwhile transfer effects of task-switching training have been proven also in other studies including samples of adolescents (Zinke et al., 2012) and young adults (Pereg et al., 2013; von Bastian and Oberauer, 2013). For instance, Zinke et al. (2012) used a similar task-switching training protocol although with less training sessions (three instead of four) and replicated a reduction of mixing but not of switching costs from pre- to post-test while far transfer effects were only found for WM updating (2-back task) and speed of processing (choice RT task). Pereg et al. (2013) used one training condition of the original Karbach and Kray (2009) study (training in task switching + verbalization + variability) and specifically tested whether this type of switching training transferred to other switching situations (cued task switching and switching after every third trial) as well as to other cognitive tasks including memory, inhibition, and choice RT tasks. Their results indicated practice-induced improvements during the training sessions as well as a larger reduction in mixing and switching costs (but only for a perceptual and not for a semantic switching task) but found no transfer to other switching situations or other cognitive variables (far transfer). Finally, the training study of von Bastian and Oberauer (2013) included the same stimuli as Karbach and Kray (2009), but used a cue-based task-switching training. They found practice-related improvements in task switching as well as near transfer to an AR-TS paradigm with bivalent stimuli, and moreover training-related improvements were correlated with near transfer gains in task switching. However, they found no evidence for far transfer to reasoning, inhibition, or working memory.

In sum, while most studies found evidence for near transfer effects the evidence for far transfer of training in task switching is mixed and less convincing, and may only occur under specific conditions that induce high demands on cognitive control and practice several executive processes at the same time, as in the original Karbach and Kray (2009) study. Indirect evidence for this view comes from a recent dual-task study by Anguera et al. (2013). In their study, they measured multitasking abilities in participants aged 20 to 79 years in a three dimensional video game and found a linear decline in dual-task performance with aging. However, older adults (60–85 years) were trained in an adaptive version of this video game for a period of 1 month. After 12 sessions of practice they showed marked improvements (compared to an active- and a no-contact control group) in multitasking performance, reaching better performance than untrained 20-year-olds. Furthermore, this improvement in multitasking was still observable after 6 months. The training also led to improvements in untrained cognitive abilities, such as enhanced sustained attention and working memory. On the basis of these findings they proposed that training in resolving interference between two tasks that occurs in dual-task like situations is most critical in order to obtain broader transfer of training in older adults.

**FIGURE 1:**
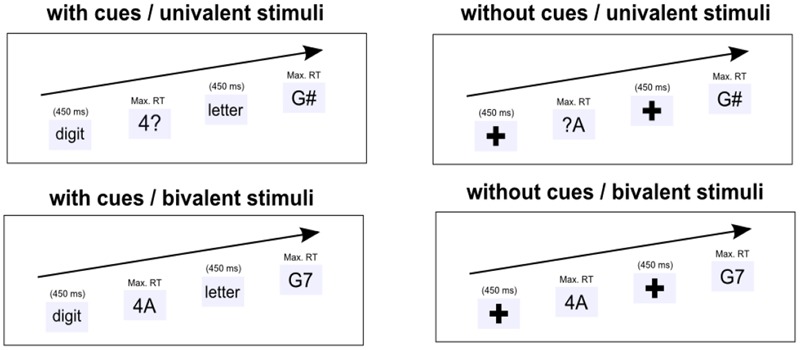
**Illustration of the trial procedure, task cues, and stimuli in the four training sessions for the four task-switching training groups.** Participants had to switch between a digit task (odd or even?) and a letter task (consonant or vowel?). Task-switching Training Group 1 received task cues and practiced with univalent stimuli (**Upper**/**left**). Task-switching Training Group 2 also received task cues and practiced with bivalent stimuli (**Lower**/**left**). Task-switching Training Group 3 practiced without task cues and with univalent stimuli (**Upper**/**right**). Task-switching Training Group 4 practiced without task cues and with bivalent stimuli (**Lower**/**right**).

Considering the recent empirical evidence, it seems that variations in the type of training condition are critical for promoting broader transfer to other cognitive abilities in older adults but maybe also in younger adults. Given that in our previous training study several components of cognitive control were practiced (as participants had to *switch* between tasks without task cues that help to *maintain* the task sequence and with ambiguous stimuli that require to *inhibit* the currently irrelevant task feature), we aimed at determining which of these control components is more critical for inducing transfer of training. To disentangle the relative involvement of switching, inhibition, and WM demands in our training, we created five different training conditions, four switching training conditions (see **Figure 1**) and one single-task training condition. First, to determine the impact of the switching component we compared the four switching groups against the single-task training group that performed the two tasks always in separate blocks throughout the training sessions. However, note that the single task group served as active control group for the switching groups but also received ambiguous stimuli and no task cues (as they were redundant). Hence, participants in this group also practiced resolving interference but not in a dual-task/switching context. We decided to include this control condition also for reasons of comparability to our first training study. Second, WM demands were manipulated by the presence or absence of task cues (see **Figure 1**). Hence, two of the switching training groups received a task cue that helped them to keep track of the task sequence (low WM demands), while the other two groups received no task cues and had to switch the task on every second trial (high WM demands). Furthermore, inhibition demands were manipulated by switching conditions in which either bivalent (ambiguous) or univalent (unambiguous) stimuli were present (see **Figure 1**). Hence, two of the switching training groups performed the task with bivalent stimuli in which the currently irrelevant task feature had to be suppressed all the time (high inhibition demands), while the other two switching training groups performed the task with univalent stimuli in which the task-relevant stimulus was combined with a neutral feature so that the stimuli directly activated the relevant task (low inhibition demands).

To examine near transfer effects and its maintenance compared to pretest performance the different switching conditions were measured in each training group at pretest, posttest, and after a 6-months follow-up measurement. Far transfer effects were measured by including cognitive tasks measuring working memory, inhibition, and context updating with several indicator tests (for details, see Materials and Methods section).

There is now evidence from a variety of studies that mixing and switching costs are substantially reduced with increasing practice in younger as well as in older adults under different type of switching conditions (for reviews, see Kray and Ferdinand, 2014). Furthermore, researchers also found that task-switching costs and age differences therein vary with the amount of task interference and memory load (Mayr, 2001). Age-related differences in task switching were more pronounced in the presence of task ambiguity (Mayr, 2001) and in the absence of task cues (Kray et al., 2002). On the basis of these finding we expected that (a) younger and older adults will show a reduction of switching costs across the four practice sessions; (b) switching costs will be larger in training conditions with high inhibition demands (with bivalent stimuli) than with low inhibition demands (with univalent stimuli); and (c) switching costs will be larger in the training conditions with high memory demands (no task cues) than with low memory demands (with task cues). We had no specific expectations about age and group differences in task-switching improvements across the four training conditions. However, most important here is to show that all groups will show switching improvements during the training session.

On the basis of our previous study, we expected near transfer of training in task switching, that is, larger performance improvements in the training groups compared to the active control group (see Karbach and Kray, 2016). However, less clear is whether the transfer of training is specifically related to the switching, working memory, or inhibition processes that differ between different variants of switching tasks. Hence, if the switching component contributes to the transfer in task switching we expect larger transfer in all training groups compared to the active control group. If the inhibition component is critical we expect larger transfer effects for the training groups that practiced with bivalent stimuli, and finally if the WM component is most critical we expect larger transfer for the groups that practiced without task cues. So far there exists less evidence for the maintenance of practice-induced improvements of task switching over a longer period of time. As training in working memory has been shown to maintain up to 6 months and longer in younger as well as in older adults (e.g., Li et al., 2008), we also expected that, if transfer effects can be observed, they persist over time.

As some researchers claimed that the training of task switching is rather specific to the trained situation (e.g., Pereg et al., 2013) the present study allows us to directly test this by assessing whether performance improvements within each of the four task-switching groups only occurs if training and transfer tasks strongly overlap in their cognitive control demands. If not, we should find transfer effects (performance gains) also in switching tasks that only partly overlap with the training task, that is, either overlap in memory or inhibition demands as those experienced at training.

In order to examine differential effects on far transfer measures we included a comprehensive cognitive test battery including indicator tests of WM span and updating, inhibition and fluid intelligence. On the basis of our previous findings (cf. Karbach and Kray, 2009) we expected to find relatively broad transfer to these measures for groups that practice all cognitive control components (switching, working memory, and inhibition), that is, for the task-switching training group that practiced the task without task cues and bivalent stimuli. We also expected that the task-switching groups that practice under higher inhibition demands may show larger transfer on inhibition measures and that the groups that practice with higher WM demands will show larger transfer on WM measures. We had no specific expectations about age differences in these effects.

## Materials and Methods

### Participants

Overall 176 participants participated for this study. All participants gave informed written consent in accordance with the protocols approved by Saarland University. They were recruited from a subject pool at Saarland University and were paid around 60 Euros to participate in the six sessions of the study, plus 20 Euros for a follow-up assessment. The study and applied methods were also approved by the local ethics committee of Saarland University. Thirteen participants had to be excluded from the analysis either because they did not want to finish the study (*n* = 9), because of health problems (*n* = 3) or because of technical problems (*n* = 1). The final sample consisted of 81 younger adults (*mean age* = 21.9 years; *age range* = 19–25 years; 49% female; Group 1: *n* = 16; Group 2: *n* = 16; Group 3: *n* = 16; Group 4: *n* = 17; Group 5: *n* = 16; see also **Table 1** and Section “Training Intervention: Training Tasks and Groups” for the description of the five groups) and 82 older adults (*mean age* = 70.8 years; age range = 65–85 years; 52% female; Group 1: *n* = 17; Group 2: *n* = 17; Group 3: *n* = 16; Group 4: *n* = 16; Group 5: *n* = 16). For the 6-months follow-up session 71 of the younger age group and 74 of the older adults were willing to return to the lab. Younger and older adults did not significantly differ in years of education (*p* = 0.11). Comprehensive information about the level of cognitive functioning is provided in the analysis of pretest performance (see Results section and **Table 1**).

**Table 1:** Means (M) and standard deviations (SD) as well as *F*- and *p*-values for training group comparisons for all pretest measures separately for the training groups and age groups.

	Training group					
	Group 1 single task – bivalent stimuli	Group 2 with cue – univalent stimuli	Group 3 with cue – bivalent stimuli	Group 4 without cue – univalent stimuli	Group 5 without cue – bivalent stimuli		
	
Age group	*M (SD)*	*M (SD)*	*M (SD)*	*M (SD)*	*M (SD)*	*F-*value	*p-*value
**Mixing costs (ms)**						
Younger	125 (96)	118 (98)	113 (80)	135 (137)	122 (76)	0.12	0.98
Older	282 (128)	272 (144)	271 (140)	278 (149)	268 (119)	0.03	0.99
**Switching costs (ms)**						
Younger	79 (53)	77 (63)	87 (53)	93 (61)	97 (57)	0.37	0.83
Older	96 (106)	106 (108)	95 (59)	108 (65)	100 (84)	0.80	0.99
**Counting span (% correct items)**						
Younger	82 (9)	82 (14)	81 (13)	82 (11)	78 (12)	0.44	0.79
Older	76 (15)	72 (12)	77 (17)	74 (13)	69 (11)	1.03	0.40
**Reading span (% correct items)**						
Younger	81 (10)	76 (14)	78 (13)	76 (18)	76 (15)	0.23	0.92
Older	74 (14)	73 (15)	73 (14)	77 (12)	72 (16)	0.55	0.70
**Digit backward (% correct items)**						
Younger	36 (9)	36 (7)	36 (11)	35 (11)	36 (8)	0.18	0.95
Older	30 (7)	25 (12)	26 (8)	22 (9)	29 (12)	1.34	0.26
**2-back (proportion of hits minus false alarms)**						
Younger	0.50 (0.3)	0.45 (0.33)	0.41 (0.3)	0.41 (0.3)	0.58 (0.2)	0.79	0.54
Older	0.41 (0.3)	0.45 (0.3)	0.40 (0.3)	0.46 (0.3)	0.46 (0.3)	0.32	0.86
**Color–Stroop Interference (ms)**						
Younger	38 (46)	27 (57)	13 (51)	37 (32)	27 (52)	0.71	0.59
Older	121 (68)	86 (67)	72 (40)	112 (140)	111 (93)	0.72	0.59
**Number–Stroop Interference (ms)**						
Younger	13 (31)	21 (44)	36 (29)	11 (37)	27 (33)	1.27	0.29
Older	6 (64)	33 (57)	4 (43)	31 (46)	48 (64)	1.46	0.22
**AX–CPT Interference (ms)**						
Younger	75 (63)	80 (76)	141 (101)	120 (73)	125 (108)	1.84	0.13
Older	123 (117)	132 (123)	114 (100)	129 (177)	96 (251)	0.13	0.97
**Raven (number correct items)**						
Younger	11 (2.43)	11 (3.19)	11 (2.16)	12 (2.37)	12 (2.66)	0.37	0.83
Older	5.3 (2.32)	5.3 (2.22)	5.3 (2.26)	4.7 (2.68)	4.9 (2.55)	0.19	0.94

### Procedure

Practice and transfer effects of the task-switching training were assessed by means of a pretest-training-posttest follow-up design. Before practice, all participants completed a pretest assessment to measure baseline performance in several cognitive tasks that lasted about 2.5 to 3 h. During pretest, all participants first gave informed consent before they filled out a demographic questionnaire. Then, we measured baseline performance in the single tasks and four different switching conditions (see **Table 1**) before subjects received a comprehensive cognitive test battery. The four training sessions were identical in structure and intensity with the previous task-switching training study (Karbach and Kray, 2009). Each training session lasted between 30 and 40 min. Testing time was shorter as compared to this previous study although participants received the same number of trials, but we shortened the preparation time on each trial in the present study. To examine transfer of the task-switching training, participants were assessed with similar type of cognitive tests and questionnaires, except the demographic questionnaire, but in contrast to the previous training study we applied parallel versions of each test and questionnaire in the posttest and follow-up sessions.

The time between pretest and posttest was not significantly different across the five training groups (*M* = 22 days; *SD* = 5.83), neither in the younger age group, *p* = 0.42, nor in the older age group, *p* = 0.14. Training sessions were restricted to be twice weekly. The time between the posttest and the follow-up session was on average 200 days (*SD* = 48.79), and again did not significantly differ between the training groups in neither of the two age groups (*p* = 0.41, *p* = 0.78, respectively).

### Measures at Pretest, Posttest, and Follow-up

#### Measurement of Near Transfer

In order to measure near transfer of task switching we assessed the baseline performance in the five different switching conditions (described above) with untrained tasks that were structurally quite similar to the training tasks. That means, the structure of tasks, the trial procedure, and block design was identical to the training conditions. We also used combinations of digits and letters but this time subjects had to perform two different tasks. In the “digit” task (Task A) participants were to decide whether the value is smaller (1, 2, 3, 4) or larger (6, 7, 8, 9) than five, and in the “letter” task (Task B) participants were to indicate whether letters were printed either in lowercase (f, t, d, j) or in uppercase (F, T, D, J).

All participants started with two practice blocks, each consisting of nine trials, in which participants performed task A or task B to make sure that they have understood the task instructions. Then, they performed six single-task blocks of task A and task B, each consisting of 17 trials. Thereafter, they performed 12 mixed-task blocks. Hence, participants performed three blocks of each of the four different switching conditions (see **Figure 1**) that were given in a constant order across subjects at each of the three measurement points: mixed blocks with cues and univalent stimuli; mixed blocks with cues and bivalent stimuli; mixed blocks without cues and univalent stimuli; and mixed blocks without cues and bivalent stimuli. Stimulus presentation was randomly selected in at each measurement time. Mixing costs were defined as the difference in performance between mixed-task blocks and single-task blocks. Switching costs were defined as difference in performance between switch trials and non-switch trials within mixed-task blocks.

#### Measurement of Far Transfer

The cognitive test battery included two or three tests and tasks of four different constructs: (1) Verbal WM was assessed by the Digit Backward, Reading Span, and Counting Span tests; (2) WM updating by the 2-back, 3-back; and (3) inhibition by the Color and Number Stroop tests and the AX-CPT (AX-Continuous Performance Task) test, and (4) fluid intelligence by Raven’s Progressive Matrices tests. In addition, as a control variable, processing speed was measured by the Digit-Symbol Substitution test. The cognitive battery included partly similar tasks and tests that have been used in a previous study (Karbach and Kray, 2009), but also new tests. Importantly, in this study were used parallel test versions of each span tasks and fluid intelligences tests, in contrast to previous training studies. For the experimental tasks we used the identical stimuli but randomly created new item lists for each measurement time.

In the *Digit Backward test* the experimenter read aloud a list of numbers of varying length (range = 2–14 items) and the participants had to repeat the numbers of each list in reverse order (adapted from Wechsler, 1981). Four lists of each length were given. The test score was the number of totally correct recalled numbers in each list. The parallel versions for the posttest and the follow-up measurements were identical except that other numbers were randomly assigned to each list.

The *Reading and Counting Span tests* were originally constructed by Kane et al. (2004), but shortened with 8 trials instead of 12 trials (cf. Karbach and Kray, 2009). The test score in each task version was the number of totally correct items. For the parallel measurements were created new item lists while the structure remained identical.

The *2-back and 3-back tasks* (adapted from McElree, 2001) was applied in which participants saw a numbers (ranging from 1 to 9) successively presented for 1000 ms. The task was to monitor the numbers and press a button if the given number was the same as two or three before, respectively, or another button in the other case. The task started with a practice block of 20 trials followed by the experimental block of 108 trials. The test score was hits minus false alarms. For the older participants an extra practice block was included with a longer stimulus presentation time of 2000 ms. to make them better familiar with the task. However, as the 3-back turned out to be too difficult for older adults they only received the 2-back task.

The *Color and Number Stroop tasks* were adapted from Salthouse and Meinz (1995). In the color version participants were presented words (e.g., ‘red’, ‘hat’) in different colors (red, blue, green, yellow). The task was to indicate the color in which the word was written. In the number version participants were presented characters (e.g., ‘3’, ‘M’) that varied in the number of the same character ranging from 1 to 4 (e.g., 3, 33, 333, 3333). Responses in both versions were given manual by pressing the left and right index and middle finger. The stimulus-response assignment was constant across participants. The task of the participants was to indicate how many characters were displayed on the screen. Interference effect was defined by subtracting mean reaction times of incongruent trials (e.g., ‘red’ in blue color; ‘3’) from the mean reaction times of neutral trials (e.g., ‘hat’; ‘M’). Parallel test versions were structurally identical but different in the item lists across the measurement times.

A modified AX – Continuous Performance Test (i.e., AX-CPT, adapted from Servan-Schreiber et al., 1996) was used to measure interference control. Participants first saw a cue (A, F, G, S) for 500 ms that was followed by a probe (X, C, M, U) for 500 ms. The probe was present until the response was given with a maximum response deadline of 1300 ms. The cue-probe interval was 2000 ms. The task was to press the right response key for an AX cue-probe combination (the frequency of which were 70% of all trials), and the left response key for each other cue-probe combination (that is: AY, BX, BY; the frequency for each type were 10%). As AY and BX trials overlapped with one element of the target pair (AX), either the cue or the probe, these trials induce interference as compared to AX and BY trials (cf. Paxton et al., 2006). Hence, we defined interference costs as the difference in mean performance between interference and non-interference trials.

As reasoning tests we applied the Raven’s Standard Progressive Matrices (for details, see Raven, 1988). The task of the participants was to find out which figure would fit best a pattern of figures from a given array. The test score was the sum of correctly solved items within 10 min. Parallel test versions were structurally identical but different in the items lists across the measurement times.

##### Training Intervention: Training Tasks and Groups

Participants of all groups were instructed to perform two tasks during the four practice sessions. As stimuli and tasks we used the original Rogers and Monsell (1995) materials in order to manipulate the amount of stimulus-induced interference namely digit-letter combinations (e.g., A4). In the one task, the *digit task* they pressed a left response key if the digit was odd (i.e., 1, 3, 5, 7) and the right response key if the digit was even (i.e., 1, 3, 5, 7). In the second task, the *letter task* they pressed the left key if the letter was consonant (i.e., G, K, M, R) and the right key if the letter was a vowel (i.e., A, E, U, I). The response assignment remained constant across the practice sessions and individuals. Small signs over the response keys helped the participants to remember the response assignments.

Training sessions for all groups consisted of 24 experimental blocks (17 trials per block), so that all participants received 1632 training trials. Mixed blocks for the task-switching groups were designed in a way that participants received an equal number of trial types (switch and non-switch), task types (A and B), and response types (left and right) and single-task blocks consisted of an equal number of task types and response types. Trials started with a cue or fixation cross that remained for 1000 ms, which was followed by the target that remained on the screen until the subject responded. The time interval between the response and the next trial was fixed to a 25 ms blank screen (see **Figure 1**). Participants were instructed to respond as fast and as accurate as possible. Feedback about their performance (error rate, RT) was given at the end of each block.

Before the training sessions, participants were assigned to one of five training groups based on their pretest performance in task switching (median RTs for single tasks and mixing costs, perceptual speed of processing, and number of correct answers), performance in the Stroop (median RT for interference costs for the color and the number task), WM span tasks (% correct answers), Updating tasks (PR scores and median RT on the AX-CPT), and the Raven score (number of correct answers). After pretest we calculated all test scores for each participant. For each age group, the first five participants were assigned randomly to the five different training groups. Then we calculated standard deviations separately for all test scores of these five participants. The sum of standard deviations served as an indicator of how similar or different these five groups were to each other. For each next participant, we tested how the indicator would change for the five potential assignments. We then selected the group for which the changing indicator score was lowest.

The training groups differed regarding the switching demands (performing single tasks versus performing mixed-task blocks), WM demands (performing mixed-task blocks with task cue versus no task cue), and inhibition demands (mixing blocks consisting of bivalent versus univalent stimuli) as described in more detail in the following (see also **Figure 1**).

*Single-task Training Group (active control group)*. In this group participants performed the letter and digit task in separate blocks (i.e., single-task blocks) that were grouped together. In each of the four practice sessions they either first practiced the letter task and then the digit task, and vice versa in the next practice session. All stimuli were bivalent, that is, participants received only digit-letter combinations (i.e., A4, 2G; U7, etc.) throughout the practice sessions. Note that this condition was similar to our previous active control group condition (Karbach and Kray, 2009) except that we used other stimulus materials (pictures instead of letter-number combinations).

*Task-switching Training Group 1 (low WM and low inhibition demands).* Like all other task-switching training groups, participants in this group received only mixed-task blocks and were instructed to switch the task on every second trial. Demands on keeping track of the task sequence were low in this group, as they received additional task cues on each trial, either the word “letter” or “digit,” indicating the next task. Also, demands on interference control were low as all stimuli were univalent, that is, the digit or letter stimuli were combined with task-irrelevant (neutral) features (i.e., [^∗^, ?, #, %]; see also **Figure 1**).

*Task-switching Training Group 2 (low WM and high inhibition demands).* Like the task-switching training group 1, participants alternated between the two tasks and received task cues in order to keep track of the task sequence. Interference demands were higher as compared to the first task-switching training group as they only received bivalent stimuli throughout the practice sessions (see **Figure 1**).

*Task-switching Training Group 3 (high WM and low inhibition demands).* Like task-switching training group 1, participants alternated between the two tasks and received only univalent stimuli. In contrast to group 1, they received no additional task cues that helped them to keep track of the task sequence. Instead they only saw a fixation cross at the beginning of each trial (see **Figure 1**).

*Task-switching Group 4 (high WM and high inhibition demands).* This training group comes closest to one of our training groups of the previous study (Karbach and Kray, 2009) in which participants had to switch between the two tasks without receiving task cues while task interference was high due to bivalent stimuli.

### Data Analysis

For the task-switching data the first trials of each block were discarded during analysis, as well as responses slower than three standard deviations from the mean of each experimental condition. For all analyses IBM SPSS 22 Statistics were used. In the Results Section for the task-switching data, we will focus on RTs, as there were no significant interaction with the factor Training Group for error rates. Mixing and switching costs were defined by two orthogonal contrasts. In the first contrast performance of single task trials were compared with non-switch and switch trials in mixed blocks (i.e., -2 1 1, mixing costs). In the second contrast performance within mixed blocks were compared between non-switch and switch trials (i.e., 0 -1 1, switching costs). Thereby, mixing and switching costs are statistically independent of each other. As baseline differences in reaction times between younger and older adults can be substantial, when comparing performance costs between younger and older adults, we also analyzed the data on the basis of log-transformed reaction times that are less sensitive to group differences in baseline performance (e.g., Kray and Lindenberger, 2000; Karbach, 2008).

The advantage here is that mean differences between log-transformed RTs correspond to ratio scores (cf. Meiran, 1996) so that the interpretation of age differences, practice and transfer effects are based on relative changes instead of absolute changes. Unless reported otherwise, results were consistent with untransformed RTs. Testing for homogeneity of variance-covariance matrices was assessed by Box’s M tests. In case of violation of assumptions, Greenhouse-Geisser corrected *p*-values are reported.

For the evaluation of transfer effects, we also calculated Cohen’s *d* or the standardized mean difference in performance between pretest and posttests (Verhaeghen et al., 1992). That is, the pretest-posttest differences (for each of the two groups) were divided by the pooled standard deviation for test occasions. We then corrected all *d*-values for small sample bias using the Hedges and Olkin correction factor (*d’*) (Hedges and Olkin, 1985).

## Results

The results section consists of four parts. In the first part, we analyzed baseline differences between the training groups for all variables of interest. In the second part, we analyzed age and training group differences in the practice effects in the training phase. In the third and fourth part, we analyzed whether near and far transfer effects, respectively, varied across age and training groups.

### Group Differences in Baseline Performance

At first we assessed whether there were baseline differences in the pretest measurement of the dependent variables of interest for near and far transfer measures between the five training groups (see **Table 1**). Therefore, pretest data were submitted to separate analyses of variance (ANOVA) for each indicator test with the between-subjects factors Age Group (younger adults/older adults) and Training Group (1/2/3/4/5). Neither the main effect for Training Group for the younger or the older adults (see **Table 1**, all *p*’s > 0.13), nor the interaction with Age Group reached significance with dependent variables of interests, indicating no baseline differences.

### Age and Training Group Differences in Training Performance

**FIGURE 2:**
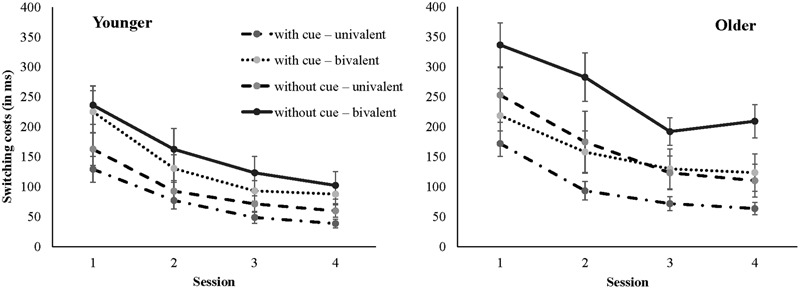
**Switching costs (in ms) as a function of session and task-switching training group separately for younger adults **(Left)** and older adults (Right)**.

To demonstrate training gains we analyzed practice-induced reductions in switching costs across the two age groups and the four task-switching training groups. Given that we had no specific hypotheses regarding differences in training curves across the four training sessions we focused the analyses on comparisons between the performance in the first and fourth training session. Mean reaction times for all experimental variables that entered the ANOVA as well as switching costs and their reduction are shown in **Table 2**, separately for the four training groups and two age groups. In addition, the reduction of switching costs across the four sessions in the four training groups is displayed in **Figure 2** separately for younger and older adults.

**Table 2:** Mean reaction times (ms) and (SD) as a function of Session (1, 4), Trial type (non-switch, switch), and Training group (Group 2, Group 3, Group 4, Group 5) separately for each Age group (younger adults, older adults) as well as switching costs, practice gains, and improvements of relative switching costs.

	Session 1						Session 4									
	Non-switch		Switch		Switching costs		Non-switch		Switch		Switching costs		Practice gains		Relative improvement	
Training group	*M*	*(SD)*	*M*	*(SD)*	*M*	*(SD)*	*M*	*(SD)*	*M*	*(SD)*	*M*	*(SD)*	*M*	*(SD)*	*M*	*(SD)*
**Younger adults**												
Group 2	592	(63)	721	(122)	129	(86)	501	(52)	540	(70)	38	(28)	91	(65)	14.1%	(0.10)
Group 3	724	(94)	950	(210)	225	(142)	565	(79)	652	(121)	88	(65)	138	(95)	15.1%	(0.9)
Group 4	595	(129)	757	(211)	163	(113)	514	(81)	574	(111)	60	(43)	103	(77)	15.2%	(0.10)
Group 5	703	(122)	939	(222)	236	(129)	564	(78)	667	(159)	102	(92)	134	(72)	15.8%	(0.10)
**Older adults**												
Group 2	848	(181)	1020	(244)	172	(87)	651	(93)	715	(111)	64	(43)	108	(79)	10.1%	(0.07)
Group 3	1110	(280)	1329	(373)	219	(181)	855	(209)	979	(267)	124	(124)	95	(102)	5.4%	(0.09)
Group 4	866	(198)	1119	(329)	253	(182)	688	(123)	798	(221)	110	(110)	143	(111)	13.8%	(0.13)
Group 5	1104	(223)	1440	(247)	337	(147)	847	(154)	1056	(243)	209	(111)	127	(143)	7.8%	(0.14)

*Group 2 = Task-switching training with task cues and univalent stimuli; Group 3 = Task-switching training with task cues and bivalent stimuli; Group 4 = Task-switching training without cues and univalent stimuli; Group 5 = Task-switching training without cues and bivalent stimuli.*

Training data were submitted to a four-way ANOVA including the within-subjects factors Session (1, 4) and Trial Type (switch, non-switch) and the between-subjects factors Age Group (younger adults, older adults) and Training Group (Group 2, Group 3, Group 4, Group 5). For the factor Training Group, we pre-specified three *a priori* contrasts according to our predictions. In the first contrast, we compared the performance between task-switching groups that practiced with univalent stimuli versus bivalent stimuli (Training Group Contrast 1). In the second contrast, we compared the performance between groups that received *univalent* stimuli and practiced with task cues versus without task cues (Training Group Contrast 2). Finally, in the third contrast we compared the performance between groups that received *bivalent* stimuli and practiced with task cues versus without task cues (Training Group Contrast 3).

The results indicated a main effect of Age Group, *F*(1,128) = 92.94, *p* < 0.001, η^2^ = 0.42, suggesting that older responded slower than younger adults. There were also main effects of Session, *F*(1,129) = 662.72, *p* < 0.001, η^2^ = 0.84, and Trial Type, *F*(1,129) = 435.71, *p* < 0.001, η^2^ = 0.77, as well as a reliable interaction between both, [Session × Trial Type: *F*(1,129) = 183.51, *p* < 0.001, η^2^ = 0.59], indicating the switching costs were reduced from the first to the fourth training session. Overall, this reduction was about 116 ms for younger adults and 118 ms for older adults (see **Table 2**), suggesting that younger as well as older adults showed large practice-related improvements in task switching (see also **Figure 2**).

Of most interest in the present study were effects of the training group conditions and their interactions with task switching and practice. Therefore, we only report significant effects of the corresponding interactions. As can be seen in **Figure 2**, the magnitude of switching costs varied across the task-switching training groups. Groups that practiced with bivalent stimuli showed larger switching costs than groups with univalent stimuli, [Trial Type × Training Group Contrast 1: *F*(1,122) = 8.28, *p* < 0.05, η^2^ = 0.06]. Comparing the two training groups that received bivalent stimuli the group that practiced without task cues showed larger switching costs than the group with task cues [Trial Type × Training Group Contrast 3: *F*(1,122) = 5.98, *p* < 0.05, η^2^ = 0.05]. Switching costs did not significantly differ between the two groups that received univalent stimuli (*p* = 0.57). All of these effects were not modulated by practice as the interactions between Session, Trial Type, and Training Group contrasts were non-significant (all *p*’s > 0.11). Also, the four-way interactions between Session, Trial Type, Training Group, and Age Group did not reach significance (all *p*’s > 0.08).

In sum, as expected younger and older benefitted from practice in task switching in all four task-switching training groups and only the magnitude of switching costs varied across the training conditions. Switching costs were greatest with high demands on cognitive control induced by task uncertainty, that is, with the presence of ambiguous stimuli and the absence of task cues.

### Near Transfer Gains and Its Maintenance

**FIGURE 3:**
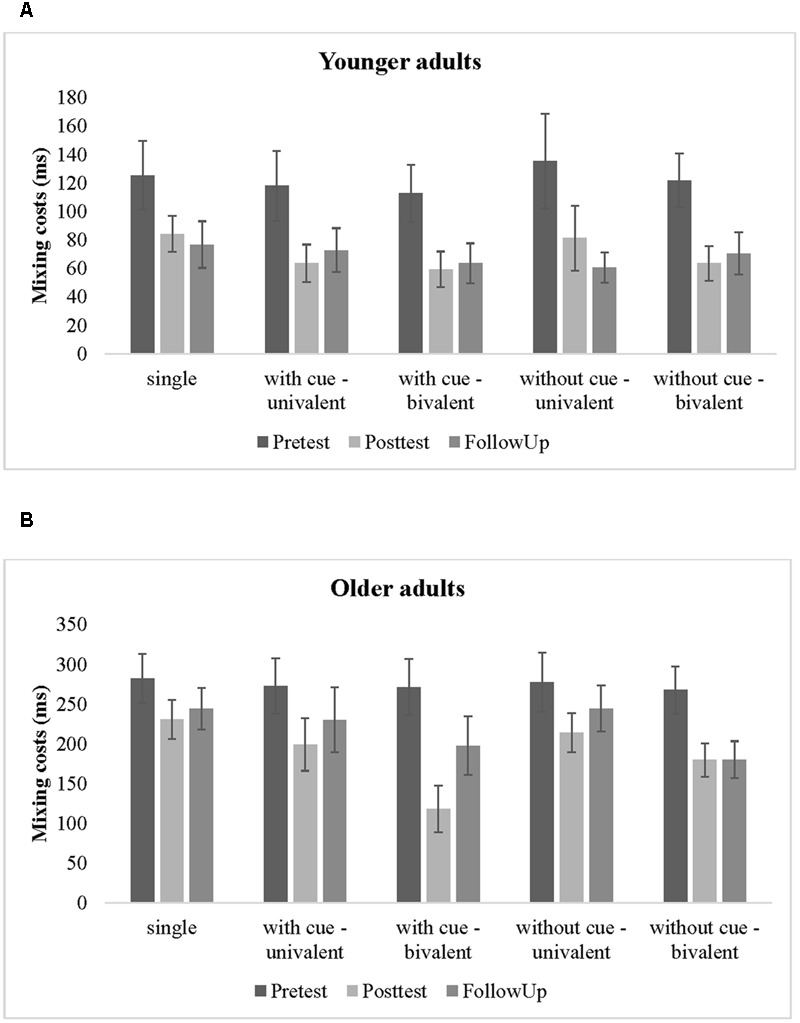
**Reduction of mixing costs from pretest to posttest to follow up (A)** for younger adults and **(B)** for older adults.

First, we analyzed age differences in the overall near transfer gains, that is, the overall improvements in task switching for the five training groups. Mean reaction times for all experimental variables and training groups are shown separately for younger and older adults in **Tables 3, 4**, respectively. Moreover, the reduction of mixing costs from pretest to posttest is displayed in **Figure 3A** for younger adults and in **Figure 3B** for older adults.

**Table 3:** Mean (M) reaction times and standard deviations (SD) for each trial type (single, non-switch, switch) as well as mixing and switching costs for younger adults separately for each training group at pretest, posttest, and follow-up.

	Training group				
	Group 1 single – bivalent	Group 2 with cue – univalent	Group 3 with cue – bivalent	Group 4 without cue – univalent	Group 5 without cue – bivalent
	
Trial type	*M (SD)*	*M (SD)*	*M (SD)*	*M (SD)*	*M (SD)*
**Pretest**					
Single	511 (49)	519 (47)	521 (71)	513 (78)	525 (58)
Non-switch	597 (100)	599 (106)	590 (115)	602 (182)	599 (99)
Switch	676 (139)	676 (150)	677 (135)	695 (231)	696 (134)
Mixing costs	125 (96)	118 (98)	113 (80)	135 (137)	122 (76)
Switching costs	79 (53)	77 (63)	87 (53)	93 (61)	97 (57)
**Posttest**					
Single	469 (45)	472 (44)	474 (66)	481 (68)	480 (48)
Non-switch	523 (55)	509 (68)	501 (77)	526 (124)	514 (61)
Switch	582 (87)	561 (107)	566 (101)	598 (181)	574 (98)
Mixing costs	84 (50)	64 (53)	59 (50)	81 (94)	64 (49)
Switching costs	59 (46)	52 (44)	65 (35)	72 (64)	60 (42)
**Follow-up**					
Single	465 (29)	474 (44)	443 (33)	466 (51)	492 (40)
Non-switch	516 (56)	525 (77)	486 (62)	499 (77)	528 (64)
Switch	568 (96)	569 (105)	527 (72)	555 (102)	597 (114)
Mixing costs	77 (63)	73 (58)	64 (46)	61 (43)	71 (57)
Switching costs	53 (53)	45 (34)	41 (22)	56 (38)	69 (60)

**Table 4:** Mean (M) reaction times and standard deviations (SD) for each trial type (single, non-switch, switch) as well as mixing and switching costs for older adults separately for each training group at pretest, posttest, and follow up.

	Training group				
	Group 1 single – bivalent	Group 2 with cue – univalent	Group 3 with cue – bivalent	Group 4 without cue – univalent	Group 5 without cue – bivalent
	
Trial type	*M (SD)*	*M (SD)*	*M (SD)*	*M (SD)*	*M (SD)*
**Pretest**					
Single	680 (68)	721 (138)	659 (102)	701 (79)	689 (64)
Non-switch	913 (155)	940 (231)	883 (179)	925 (176)	907 (141)
Switch	1010 (199)	1047 (292)	978 (203)	1033 (204)	1007 (151)
Mixing costs	282 (128)	272 (144)	271 (140)	278 (149)	268 (119)
Switching costs	96 (106)	106 (108)	95 (59)	108 (65)	100 (84)
**Posttest**					
Single	613 (70)	678 (108)	671 (110)	669 (84)	660 (79)
Non-switch	787 (128)	829 (204)	744 (159)	832 (151)	782 (132)
Switch	902 (157)	926 (236)	835 (202)	934 (194)	897 (146)
Mixing costs	231 (101)	199 (137)	118 (117)	214 (98)	180 (83)
Switching costs	115 (66)	97 (72)	91 (71)	102 (71)	115 (84)
**Follow-up**					
Single	635 (88)	694 (150)	641 (80)	700 (117)	671 (80)
Non-switch	821 (134)	874 (236)	784 (172)	891 (170)	789 (126)
Switch	936 (165)	975 (275)	893 (228)	998 (182)	913 (154)
Mixing costs	244 (97)	230 (163)	198 (142)	244 (104)	180 (93)
Switching costs	115 (96)	101 (78)	109 (78)	107 (41)	123 (74)

Data were submitted to a four-way ANOVA including the within-subjects factors Session (pretest, posttest) and Trial Type (single, non-switch, switch) and the between-subjects factors Age Group (younger adults, older adults) and Training Group (Group 1, Group 2, Group 3, Group 4, Group 5). For the factor Training Group we pre-specified four *a priori* contrasts according to our predictions. In the first contrast we compared the performance between the single-task group and the task-switching groups (Training Group Contrast 1). In the second contrast we compared the performance between task-switching groups that received *univalent stimuli* and task-switching groups that received *bivalent stimuli* (Training Group Contrast 2). In the third contrast, we compared the performance between the group that received *univalent stimuli with* task cues and the group that received *univalent stimuli without* task cues (Training Group Contrast 3). In the fourth contrast, we compared the performance between the group that received *bivalent stimuli with* task cues and the group that received *bivalent stimuli without* task cues (Training Group Contrast 4).

According to our predictions we focus on the interactions with training group. Here, we found that switching costs did not change differently from pretest to posttest across the training groups (*p* = 0.84), but mixing costs changed differently from pretest to posttest across the training groups [Session × Trial Type Contrast 1 × Training Group: *F*(4,158) = 3.55, *p* < 0.05, η^2^ = 0.08], and this effect was further modulated by age in tendency [Session × Trial Type Contrast 1 × Training Group × Age Group: *F*(4,158) = 2.20, *p* = 0.07, η^2^ = 0.05] (see also **Figures 3A,B**). The first training group contrast indicated a larger reduction of mixing costs from pretest to posttest for the task-switching training groups than for the single-task group [Session × Trial Type Contrast 1 × Training Group Contrast 1: *F*(1,154) = 6.33, *p* < 0.05, η^2^ = 0.04], and this effect was no further modulated by age (*p* > 0.26). For the second training group contrast was also significant [Session × Trial Type Contrast 1 × Training Group Contrast 2: *F*(1,154) = 5.10, *p* < 0.05, η^2^ = 0.03] and this time the effect was further modulated by age [Session × Trial Type Contrast 1 × Training Group Contrast 1 × Age Group: *F*(1,154) = 4.02, *p* < 0.05, η^2^ = 0.03]. Therefore, we run separate ANOVAs for each age group. A larger reduction of mixing costs for the bivalent than for the univalent training groups were only found in the older age group [Session × Trial Type Contrast 1 × Training Group Contrast 2: *F*(1,77) = 7.89, *p* < 0.05, η^2^ = 0.09] but not in the younger age group (*p* = 0.85). Finally, while the reduction of mixing costs did not differ between training groups that practiced with univalent stimuli with cues and without cues (*p* = 0.82), we found a difference in the reduction of mixing costs between the two bivalent groups at least in tendency [Session × Trial Type Contrast 1 × Training Group Contrast 4: *F*(1,154) = 3.17, *p* = 0.08, η^2^ = 0.02], that was again further modulated by age [Session × Trial Type Contrast 1 × Training Group Contrast 4 × Age Group: *F*(1,154) = 4.03, *p* < 0.05, η^2^ = 0.03]. Therefore, we again run separate ANOVAs for each age group. The larger reduction of mixing costs for the bivalent group with cues than without cues was only found in the older age group [Session × Trial Type Contrast 1 × Training Group Contrast 4: *F*(1,77) = 6.19, *p* < 0.05, η^2^ = 0.07] but not in the younger age group (*p* = 0.86).

However, **Figure 3B** also shows that in the group of older adults mixing costs between the two task-switching training groups that practiced with univalent stimuli seemed to be not different from the single-task training group that practiced with bivalent stimuli. Therefore, we run a *post hoc* contrast and found that the difference between these training groups was indeed not significant (*p* = 0.66).

Second, to examine whether near transfer gains (i.e., the reduction of mixing costs) were maintained over a period of 6 months, relative to baseline performance, data were submitted to a four-way ANOVA including the within-subjects factors Session (pretest, follow-up) and Trial Type (single, non-switch, switch) and the between-subjects factors Age Group (younger adults, older adults) and Training Group (Group 1, Group 2, Group 3, Group 4, Group 5). For the factor Training Group the same four *a priori* contrasts were used as previously. The corresponding data are also plotted in **Figures 3A,B**.

The results indicated a larger reduction of mixing costs from pretest to follow up for the two task-switching training groups that practiced with bivalent than with univalent stimuli [Session × Trial Type Contrast 1 × Training Group Contrast 2: *F*(1,136) = 4.14, *p* < 0.05, η^2^ = 0.03]. However, the larger reduction of mixing costs in task-switching groups compared to the single-task training group disappeared (*p* = 0.21).

In sum, for younger adults we only found that the task-switching groups showed a larger reduction in mixing costs than the single task training group from pretest to posttest but these performance gains were not maintained over a longer period of time. In contrast, older adults showed larger gains for the two task-switching groups that practiced with bivalent than with univalent stimuli, hence for conditions with high inhibition demands, and these transfer gains, relative to initial task performance before training, were maintained over a time period of 6 months.

### Transfer Gains as a Function of Overlap to the Training Condition

**FIGURE 4:**
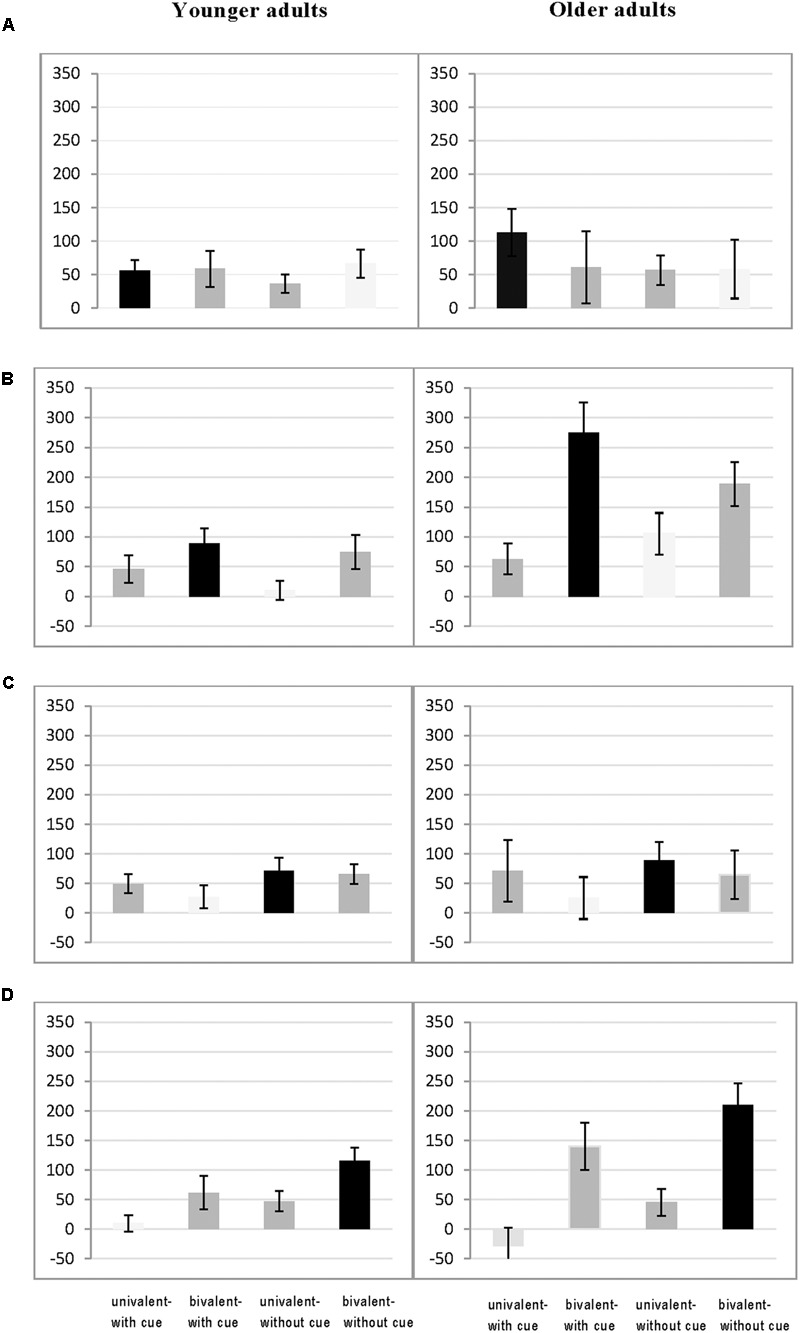
**Reduction of mixing costs from pretest to posttest separately for younger adults (left) and for older adults (right) as a function of overlap between the training and transfer condition for the Task-switching Group 1 (A)** Group 2 **(B)** Group 3 **(C)** and Group 4 **(D)**.

To further examine whether transfer gains (i.e., the reduction in mixing costs) varied as a function of overlap between training conditions and transfer condition we also analyzed age differences in transfer gains separately for each of the four task-switching training groups. The corresponding data are displayed in **Figures 4A–D**. They show that for most of the conditions transfer gains were larger for those conditions in which they were trained (highlighted by the black bars in **Figures 4A–D**) as compared to conditions in which the training shared only one feature either the cueing condition (with or without cues) or the interference condition (univalent or bivalent) (indicated by dark gray bars in **Figures 4A–D**) and smallest transfer gains are obtained for conditions that did not overlap with the two features of the training condition (see light gray bars in **Figures 4A–D**). To confirm this observation, mixing costs were submitted to an ANOVA including within-subjects factors Session (pretest, posttest) and Switching Condition (with cues/univalent, with cues bivalent, no cues/univalent, no cues/bivalent) and the between-subjects factor Age Group (younger, older) separately for each of the four task-switching training groups. We specified contrasts along to our expectation that training gains are largest for the condition that overlapped in demands on working memory and inhibition between training and transfer situation as compared to the other conditions and then we tested whether there were significant differences in gains to those conditions that overlapped either in demands on WM or inhibition between training and transfer situation.

#### Task-Switching Training Group (with Cues/Univalent)

As can be seen in **Figure 4A**, only for the older adults we found a larger reduction in mixing costs for the condition that overlapped with the training condition as compared to all other conditions [*F*(1,81) = 6.01, *p* < 0.05, η^2^ = 0.07]. We also obtained a larger reduction in mixing costs for the trained condition (with cues/univalent) as compared to the condition that only shared the univalent feature but not the cueing condition for both younger and older adults [*F*(1,162) = 18.50, *p* < 0.01, η^2^ = 0.10]. Finally, we found age differences in the reduction of mixing costs between the trained condition and the condition that shared the cueing situation (with cues) but with bivalent stimuli [*F*(1,161) = 4.28, *p* < 0.05, η^2^ = 0.03].

#### Task-Switching Training Group (with Cues/Bivalent)

For this training group we found larger reductions in mixing costs for the condition (with cues/bivalent) that corresponded to the training condition as compared to all other three conditions [*F*(1,162) = 8.95, *p* < 0.01, η^2^ = 0.05] and this effect was more pronounced in the older than in the younger adults [*F*(1,161) = 4.26, *p* < 0.05, η^2^ = 0.03]. We also obtained a larger reduction in mixing costs for the condition corresponding the training condition than the condition sharing only the cueing condition (with cues) but with univalent stimuli [*F*(1,162) = 8.53, *p* < 0.01, η^2^ = 0.05] and again this effect was more pronounced in older than in younger adults [*F*(1,161) = 4.28, *p* < 0.05, η^2^ = 0.03]. Interestingly, the reduction in mixing costs did not differ for conditions that shared bivalent stimuli (*p* = 0.86) and age differences in this comparison were absent (*p* = 0.27).

#### Task-Switching Training Group (without Cues/Univalent)

Similar to the previous training group we found a larger reduction in mixing costs for the condition (without cues/univalent) that corresponded to the training condition as compared to all other conditions [*F*(1,162) = 3.99, *p* < 0.05, η^2^ = 0.03]. Results also revealed a larger reduction in mixing costs for the condition corresponding the training condition than for the condition sharing univalent stimuli [*F*(1,162) = 8.53, *p* < 0.01, η^2^ = 0.05] but not for conditions sharing the cueing condition (*p* = 0.50). There were no significant age differences in these effects (all *p*’s > 0.11).

#### Task-Switching Training Group (without Cues/Bivalent)

Again, similar to the two previous training groups we found a larger reduction in mixing costs for the condition (without cues/bivalent) that corresponded to the training condition as compared to all other three conditions [*F*(1,162) = 11.89, *p* < 0.01, η^2^ = 0.07]. Results also revealed a larger reduction in mixing costs for the condition corresponding the training condition than for the condition sharing the cueing condition [*F*(1,162) = 18.50, *p* < 0.01, η^2^ = 0.10] but not for conditions sharing the interference condition (*p* = 0.87). Again there were no significant age differences in all of these effects (all *p*’s > 0.26).

In sum, with the exception of the first training group the results indicated that a higher overlap between the training and the transfer condition lead to larger reductions in mixing costs than compared to the other conditions. For conditions that overlap in high inhibition demands (bivalent stimuli) or in high WM demands (without cues) we did not find a difference in the amount of transfer, neither for younger nor for the older adults. Age differences were only found in the first two training groups in a way that older showed more specific transfer effects as compared to younger adults.

### Far Transfer Effects

To assess far transfer of the task-switching training to WM span and updating, inhibition and fluid intelligence measures, we first proved the correlations between the three measurement times of the parallel test versions. Correlations ranged between *r* = 0.69 and *r* = 0.70 for the Digit Backward, and between *r* = 0.40 and *r* = 0.59 for the Reading and Counting Span, between *r* = 0.42 and *r* = 0.74 for the 2-back, between *r* = 0.23 and *r* = 0.27 for the 3-back task, between *r* = 0.10 and *r* = 0.30 for the Color–Stroop interference score, between *r* = 0.26 and *r* = 0.23 for the Number–Stroop interference score, between *r* = 0.11 and *r* = 0.12 for the AX–CPT interference costs score, and between *r* = 0.83 and *r* = 0.86 for the Raven score. Hence, especially the interference costs and *n*-back measures were not very reliable across measurement times and therefore the results should be taken with caution.

The corresponding dependent variables were submitted to three-way ANOVAs, including the within-subjects factors Session (pretest, posttest) and the between-subjects factors Age Group (younger adults, older adults) and Training Group (Group 1, Group 2, Group 3, Group 4, Group 5). The corresponding data are shown in **Table 5** for the younger adults and in **Table 6** for the older adults. As can be seen in both tables, most of the variables did not change substantially from pretest to posttest. In the following, we will only report interactions of interest, such as two-way interactions between session and training group or three-way interactions between session, training group, and age group.

**Table 5:** Means (M) and standard deviations (SD) for the far transfer measures as a function of session (pretest/posttest) separately for the five training groups for the younger age group.

	Training group				
	Group 1 single task – bivalent stimuli	Group 2 with cue – univalent stimuli	Group 3 with cue – bivalent stimuli	Group 4 without cue – univalent stimuli	Group 5 without cue – bivalent stimuli
	
Session	*M (SD)*	*M (SD)*	*M (SD)*	*M (SD)*	*M (SD)*
**Counting span (% correct items)**						
Pretest	82 (9)	82 (14)	81 (13)	82 (11)	78 (12)
Posttest	83 (12)	86 (14)	80 (14)	81 (11)	80 (12)
**Reading span (% correct items)**						
Pretest	81 (10)	76 (14)	78 (13)	76 (18)	76 (15)
Posttest	77 (12)	78 (15)	78 (16)	79 (15)	82 (10)
**Digit backward (% correct items)**						
Pretest	36 (9)	36 (7)	36 (11)	35 (11)	36 (8)
Posttest	41 (9)	37 (8)	40 (9)	37 (12)	42 (16)
**2-back (proportion of hits minus false alarms)**						
Pretest	0.50 (0.26)	0.45 (0.30)	0.41 (0.26)	0.41 (0.32)	0.58 (0.19)
Posttest	0.58 (0.20)	0.52 (0.19)	0.50 (0.25)	0.45 (0.28)	0.60 (0.20)
**Color–Stroop Interference (ms)**						
Pretest	38.2 (45.9)	27.4 (57.3)	13.1 (50.7)	37.3 (31.7)	27.1 (52.2)
Posttest	27.3 (26.0)	19.4 (52.9)	36.1 (49.3)	22.7 (40.0)	15.6 (34.4)
**Number–Stroop Interference (ms)**						
Pretest	13.2 (30.6)	20.8 (44.1)	36.2 (28.8)	10.9 (37.4)	26.9 (32.9)
Posttest	15.4 (21.2)	7.9 (34.0)	12.7 (36.3)	24.2 (36.2)	12.6 (26.4)
**AX–CPT Interference (ms)**						
Pretest	75 (63)	80 (76)	141 (101)	120 (73)	125 (108)
Posttest	86 (51)	77 (69)	91 (53)	102 (65)	118 (65)
**Raven**						
Pretest	10.9 (2.4)	11.4 (3.2)	11.4 (2.2)	11.5 (2.4)	11.6 (2.7)
Posttest	11.0 (1.6)	9.8 (3.2)	11.5 (1.8)	10.0 (2.8)	10.5 (2.6)

**Table 6:** Means (M) and standard deviations (SD) for the far transfer measures as a function of session (pretest/posttest) separately for the five training groups for the older age group.

	Training group				
	Group 1 single task – bivalent stimuli	Group 2 with cue – univalent stimuli	Group 3 with cue – bivalent stimuli	Group 4 without cue – univalent stimuli	Group 5 without cue – bivalent stimuli
	
Session	*M (SD)*	*M (SD)*	*M (SD)*	*M (SD)*	*M (SD)*
**Counting span (% correct items)**						
Pretest	76 (15)	72 (12)	77 (17)	74 (13)	69 (11)
Posttest	73 (15)	76 (19)	77 (11)	81 (11)	74 (16)
**Reading span (% correct items)**						
Pretest	74 (14)	73 (15)	73 (14)	77 (12)	72 (16)
Posttest	66 (16)	71 (14)	71 (17)	71 (14)	70 (19)
**Digit backward (% correct items)**						
Pretest	30 (7)	25 (12)	26 (8)	22 (9)	29 (12)
Posttest	31 (10)	28 (11)	28 (8)	25 (9)	29 (10)
**2-back (proportion hits minus false alarms)**						
Pretest	0.41 (0.27)	0.45 (0.28)	0.40 (0.28)	0.46 (0.26)	0.46 (0.31)
Posttest	0.49 (0.25)	0.52 (0.21)	0.49 (0.22)	0.40 (0.30)	0.56 (0.15)
**Color–Stroop Interference (ms)**						
Pretest	121 (67)	85 (67)	72 (40)	112 (141)	111 (93)
Posttest	105 (104)	107 (75)	61 (42)	86 (90)	131 (77)
**Number–Stroop Interference (ms)**						
Pretest	6.15 (64)	33.4 (56)	3.70 (43)	31 (46)	48.4 (64)
Posttest	33.5 (92)	31.6 (66)	33.1 (60)	4.87 (56)	14.1 (52)
**AX–CPT Interference (ms)**						
Pretest	123 (117)	132 (123)	114 (100)	129 (177)	96 (251)
Posttest	99 (147)	123 (115)	108 (111)	154 (129)	146 (101)
**Raven**						
Pretest	5.28 (2.3)	5.27 (2.2)	5.33 (2.3)	4.69 (2.7)	4.88 (2.6)
Posttest	5.33 (2.2)	5.00 (2.1)	4.67 (1.8)	4.38 (1.2)	5.00 (2.2)

#### Working-Memory Span

For neither of the three WM variables we found significant two-way or three-way interactions (all *p*’s > 0.09). To increase the reliability of measurement we also used a composite score of all three measures by computing the mean of the three z-transformed measures. The correlations between pretest and posttest measurement of this composite measure was *r* = 0.74 for younger adults and *r* = 0.76 for older adults. Results of the ANOVA indicated that the group contrast comparing single-task and task-switching groups showed a tendency for an interaction with Session, *F*(1,154) = 2.95, *p* = 0.09, η^2^ = 0.02. We also determined the effect sizes for both age groups separately that were low and negative for the single task training groups (*d’* = -0.02 for younger adults and *d’* = -0.35 for older adults) but also small for the task-switching groups (*d’* = 0.12 for younger adults and *d’* = -0.01 for older adults). Hence, we found no evidence for improvements in WM capacity after task-switching training.

#### Working-Memory Updating

Again for neither of the WM updating measures we obtained significant two or three-way interactions (all *p*’s > 0.19). Again to increase the reliability of measurement we computed composite scores, that is, means of the z-transformed scores of the 2-back and 3-back task (only for younger adults). The group contrast comparing single-task and task-switching groups showed no significant difference in performance between pre- and post-test (*p* = 0.35).

#### Inhibition

For the three inhibition measures we found no significant two-way and three-way interactions for the Color–Stroop interference effect (all *p*’s > 0.12) and the AX–CPT interference score (all *p*’s > 0.19). Only for the Number–Stroop interference effect we found an interaction between session and the group contrast comparing the single task training group with all task-switching training groups, *F*(1,158) = 3.89, *p* = 0.05, η^2^ = 0.02, indicating a larger decease in interference costs from pretest to posttest for the task-switching than the single task groups. As correlations between the three interference costs measures were rather low ranging between *r* = 0.04 and *r* = 0.20 we did not compute a composite measure for these measures.

#### Fluid Intelligence

For the performance on the Raven’s we also found no interactions between session and training group contrasts or between session, age group, and training group contrasts (all *p*’s > 0.13). There was only a tendency for an interaction between session and the training contrast comparing the single with all task-switching groups, *F*(1,153) = 3.40, *p* = 0.07, η^2^ = 0.02. However, as can be seen in **Tables 5, 6**, these changes were in opposite to expectations as Raven’s performance declined and in tendency more for the treatment groups than for the active control group. Effects sizes for the single task training groups were rather small and positive for the younger adults (*d’* = 0.08) and for the older adults (*d’* = 0.17) and negative for the task-switching training groups for the younger adults (*d’* = -0.38) as well as for the older adults (*d’* = -0.09).

## Discussion

The main goal of the present study was to systematically investigate the impact of WM and inhibition demands on improvements in task switching, its maintenance and near and far transfer effects as well as age differences therein. In particular, we aimed at identifying whether and what kind of control processes may contribute to the transfer of switching training to new switching situations and other cognitive control tasks that varied in WM and inhibition demands. To achieve these goals we created five different training conditions that varied in switching (single task versus mixing tasks), inhibition (bivalent versus univalent stimuli) and WM demands (without versus with task cues). We compared younger and older adults in transfer and maintenance effects across the different switching training conditions with a pretest-training-posttest follow-up design.

Results of this training study revealed several important new insights about which cognitive processes are critical for the transfer and maintenance of training effects in cognitive control in younger and older adults. At first, the analysis of practice data showed that our experimental manipulations were successful and lead to variations in the magnitude of switching costs. Switching costs were largest in the groups that received bivalent stimuli and no task cues (high WM demands) as compared to the groups that received task cues (low WM demands), and switching costs were larger for groups practicing with bivalent (high inhibition demands) than with univalent stimuli (low inhibition demands). Also note that for the groups receiving univalent stimuli switching costs were not different depending on whether task cues were present or not. As for univalent stimuli task cues are in principle redundant subjects may adopted a strategy to wait for the target presentation in order to select the appropriate response without advance preparation. More importantly for the interpretation of transfer effects is that all training and age groups showed a substantial reduction of switching costs throughout the four practice sessions. Effect sizes for the practice gains varied between *d’* = 1.21 and *d’* = 1.42 for the younger adults and between *d’* = 0.62 and *d’* = 1.59 for the older adults.

A second noteworthy finding is that we obtained age-differential effects in the transfer of training gains to new untrained switching situations as a function of training demands. Overall, younger adults showed a larger reduction in mixing costs from pre- to post-test after task-switching training as compared to the active control group independently of the WM and inhibition demands throughout the training. This seems to suggest that training in switching being most critical in that age group but the observed training benefit was not stable over time. One may argue that the variations in WM and inhibition demands were not different and challenging enough in this age group to induce a mismatch between training demands and actual level of cognitive functioning but the results from the practice phase clearly indicated a substantial variation in the magnitude of switching costs also in the younger age group a finding that speaks against this potential explanation. Hence, training in task switching seems to be rather narrow in scope in the group of younger adults, in line with other findings (e.g., Pereg et al., 2013). In contrast, the older adults showed larger performance improvements in task switching when they practiced task switching with bivalent stimuli instead of univalent stimuli, suggesting that inhibition demands are critical in that age group. Notably, not only inhibition counts for the elderly given that the single task training group has also received bivalent stimuli but this condition did not require to maintain both tasks and to switch between them. Indeed our analysis revealed that the active control group did not differ from the other two task-switching groups practicing with univalent stimuli. These findings suggest that control processes required for resolving task interference in dual-task like switching situations are most critical for inducing transfer effects, and moreover, we were able to show for the first time that elderly adults were able to maintain these benefits at least for 6 months. In general, our results are consistent with previous task-switching studies by showing switching improvements in a new, untrained switching situation in younger adults (Pereg et al., 2013; von Bastian and Oberauer, 2013) as well as in older adults (Bherer et al., 2005; Karbach and Kray, 2009; Anguera et al., 2013). They are also consistent with the claim by Anguera et al. (2013) that the training in resolving interference between two competing tasks (as required in dual-task and switching situations) is a key component for inducing transfer of training in older adults. The present study directly tested this idea by systematically manipulating the amount of interference between tasks while switching between them and in support of this view transfer of training was restricted to the bivalent training conditions in the elderly.

A third new finding of the present study is that the transfer of switching training depends on the amount of overlap between training and transfer situation and by this on the type of cognitive control processes practice during the training sessions. Comparing the reductions of mixing costs within the four task-switching training groups indicated - with the exception for the young group with lowest demands on cognitive control (with cues/univalent stimuli) – a general pattern of larger transfer gains with more overlap between the training and the transfer situation in younger as well as in older adults. However, reductions in mixing costs did not differ when training and transfer situation required high WM demands (without cues) or high inhibition demands (bivalent stimuli), again suggesting that transfer of training in task switching only occurs when the training situation is challenging, that is, when WM updating and interference control is required and practiced. Our findings are consistent with findings from study by von Bastian and Oberauer (2013) who found near transfer from a cued-task switching training to an uncued switching task with bivalent stimuli. In contrast, one study by Pereg et al. (2013) did not find evidence for a transfer to similar switching situations, but in this study only WM demands were varied by increasing the length of task-repetition trials (without cues) and presenting cues in a random manner. Therefore, these findings are not directly comparable to those of the present study. However, the overall findings clearly show that the amount of transfer of a switching training is strongly dependent on the overlap between training and transfer situation and which type of control processes are trained, and by this, transfer of switching training is more narrow in scope as previously assumed (cf. Karbach and Kray, 2009).

Finally, in contrast to our previous findings results of the present study did not replicate the broad transfer to other cognitive tasks, more in line with other recent task-switching studies (Zinke et al., 2012; Pereg et al., 2013; von Bastian and Oberauer, 2013). Several reasons might explain this discrepancy in findings across both studies. First, we created and applied parallel versions of each test and task. We did this in the present study in order to reduce repeated measurement effects as we had three measurement times in our training design. Although correlations between the three measurement times were moderate to high for the WM span measures and the fluid intelligence test, especially the interference costs scores were not reliable across time and also did not correlate with each other, which in turn strongly decreases the likelihood to obtain far transfer effects. Second, we changed the stimulus material in the training and transfer switching tasks as we manipulated the amount of interference (bivalent and univalent stimuli) in the present study. In contrast, in the Karbach and Kray (2009) study we used pictures that integrated features of both tasks within the same object, such as a red apple and a black- and white printed tomato, which makes it difficult to selectively attend to only one currently relevant task feature and by this increase cognitive control demands. In the present study, we have used digit-letter combinations in which features of both tasks appeared side-by-side and therefore may are easier to selectively attend to. Interestingly the only measure in which we observed far transfer effects was the Number Stroop task that overlaps with the training task in the type of stimuli (i.e., digits), pointing to stimulus-specific effects in the transfer of training in task switching in the present study. Third, during training the type of switching tasks remained the same and the demands on cognitive control were much lower in comparison to the previous study (here all training groups received bivalent stimuli), which may limited the likelihood to induce transfer effects to other cognitive tasks as well as the power to detect them. In order to reduce such stimulus-specific as well as task-specific effects further studies need to include various stimulus domains and different task sets in their training intervention in order to foster the transfer of training.

Finally, some limitations in the interpretation of our findings should be noted. First, we only reported relative improvements from the first to the forth practice session, ignoring potential age and/or group differences in learning curves. However, most important here was to show that we found the expected effects of our experimental manipulation namely differences in switching costs as a function of demands on inhibition and working memory and that we obtained improvements on switching for all groups. Second, given the complexity of our training design even a sample size of 16 participants in each group may was insufficient to detect smaller effects of experimental manipulations especially for the follow-up results that were based on an even smaller n for each group. Third, one may argue that a training intervention with only four practice sessions was rather short (as compared to some other training studies) and that such short interventions are unlikely to induce prolonged cognitive plasticity. We did not apply a longer practice phase to better compare our results to previous findings. Notably in this respect is, however, that it has also been shown that very intense training interventions sometimes lead to a lack of motivation and results in less transfer (cf. Toril et al., 2014). Finally, although we argue for low reliabilities of the far transfer measures across the three measurement times as a potential source for the lack of far transfer effects in the present study it is also conceivable that low reliability is caused by inter-individual variability of training effects in these measures. However, as we have no information about the parallel test reliabilities of these measures at pretest from an independent sample we cannot finally conclude on the reasons for a failure in far transfer. Hence, this point has to be carefully considered in future training research.

To summarize and conclude, differential cognitive control components are critical for inducing transfer of training in task switching in younger and older adults. While for younger adults practice in switching leads to larger transfer gains independently of inhibition and WM demands, older adults strongly profit from practice in resolving interference between two competing tasks. Our findings also indicate that transfer gains vary with the degree of overlap between training and transfer tasks and by this with the type of control processes involved. Hence transfer of training is possible when the training is challenging but it is also specific to the trained processes.

## Author Contributions

Both authors were involved in planning the study design. Analysis of data and their presentation was conducted by FB and JK wrote the most parts of the paper.

## Conflict of Interest Statement

The authors declare that the research was conducted in the absence of any commercial or financial relationships that could be construed as a potential conflict of interest.
